# Beyond collective intelligence: Collective adaptation

**DOI:** 10.1098/rsif.2022.0736

**Published:** 2023-03-22

**Authors:** Mirta Galesic, Daniel Barkoczi, Andrew M. Berdahl, Dora Biro, Giuseppe Carbone, Ilaria Giannoccaro, Robert L. Goldstone, Cleotilde Gonzalez, Anne Kandler, Albert B. Kao, Rachel Kendal, Michelle Kline, Eun Lee, Giovanni Francesco Massari, Alex Mesoudi, Henrik Olsson, Niccolo Pescetelli, Sabina J. Sloman, Paul E. Smaldino, Daniel L. Stein

**Affiliations:** ^1^ Santa Fe Institute, Santa Fe, NM 87501, USA; ^2^ Complexity Science Hub Vienna, 1080 Vienna, Austria; ^3^ Vermont Complex Systems Center, University of Vermont, Burlington, VM 05405, USA; ^4^ South Denmark University, Odense 5230, Denmark; ^5^ School of Aquatic and Fishery Sciences, University of Washington, Seattle, WA 98195, USA; ^6^ Department of Zoology, University of Oxford, Oxford OX1 3PS, UK; ^7^ Department of Mechanics, Mathematics and Management, Politecnico di Bari, Bari 70125, Italy; ^8^ Psychological and Brain Sciences, Indiana University, Bloomington, IN 47405, USA; ^9^ Department of Social and Decision Sciences, Carnegie Mellon University, Pittsburgh, PA 15213, USA; ^10^ Department of Mathematics, Max-Planck-Institute for Evolutionary Anthropology, Leipzig 04103, Germany; ^11^ Biology Department, University of Massachusetts Boston, Boston, MA 02125, USA; ^12^ Centre for Coevolution of Biology and Culture, Durham University, Anthropology Department, Durham, DH1 3LE, UK; ^13^ Centre for Culture and Evolution, Division of Psychology, Brunel University London, Uxbridge, UB8 3PH, UK; ^14^ Department of Scientific Computing, Pukyong National University, 45 Yongso-ro, Nam-gu, Busan, 48513, Republic of Korea; ^15^ Department of Ecology and Conservation, University of Exeter, Penryn TR10 9FE, UK; ^16^ New Jersey Institute of Technology, Newark, NJ 07102, USA; ^17^ Department of Computer Science, University of Manchester, Manchester, M13 9PL, UK; ^18^ Department of Cognitive and Information Sciences, University of California, Merced, CA 95343, USA; ^19^ Department of Physics and Courant Institute of Mathematical Sciences, New York University, New York, NY 10012, USA

**Keywords:** collective adaptation, collective intelligence, social networks, social cognition, computational models

## Abstract

We develop a conceptual framework for studying collective adaptation in complex socio-cognitive systems, driven by dynamic interactions of social integration strategies, social environments and problem structures. Going beyond searching for ‘intelligent’ collectives, we integrate research from different disciplines and outline modelling approaches that can be used to begin answering questions such as why collectives sometimes fail to reach seemingly obvious solutions, how they change their strategies and network structures in response to different problems and how we can anticipate and perhaps change future harmful societal trajectories. We discuss the importance of considering path dependence, lack of optimization and collective myopia to understand the sometimes counterintuitive outcomes of collective adaptation. We call for a transdisciplinary, quantitative and societally useful social science that can help us to understand our rapidly changing and ever more complex societies, avoid collective disasters and reach the full potential of our ability to organize in adaptive collectives.

## 1. Introduction

Human life is universally structured by assortment into collectives—groupings of various sizes and permanence, from small groups and teams to large organizations and communities [1]. Collectives shape and are shaped by individual cognitions, patterns of interactions, and the problem structures they encounter and create [2]. The rapidly increasing scale and complexity of our collectives potentially magnifies threats to our societies which are difficult to understand and predict [3], including the spread of conspiracy theories [4], denial of facts [5,6], extreme polarization [7] and violent extremism [8].

**Figure 1.**
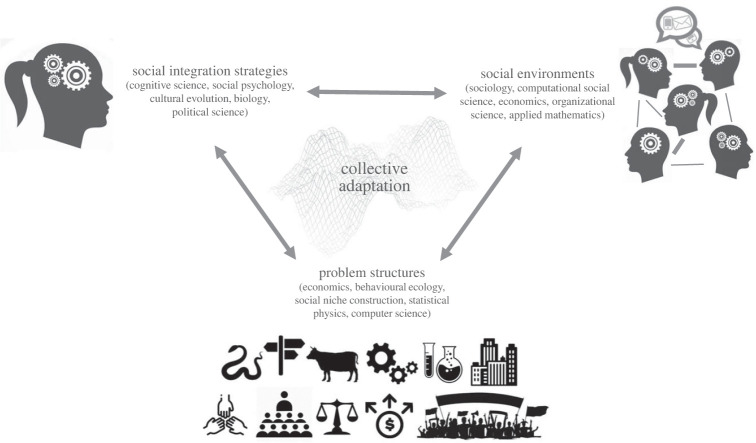
Collective adaptation can be seen as an emergent property of a complex socio-cognitive system driven by dynamic interactions of social integration strategies, social environments and problem structures collectives face. Collectives take different trajectories when navigating the complex adaptive landscape of these interactions. The system components are wholly entangled, but each has been studied in relatively isolated disciplines, and extant models of collective dynamics rarely include all components. Combining relevant findings and methods in different disciplines (box 1) is critical for understanding collective adaptation, avoiding parallel efforts (box 2) and building quantitative models that enable answering important outstanding theoretical and practical questions about human sociality.

**Figure 2.**
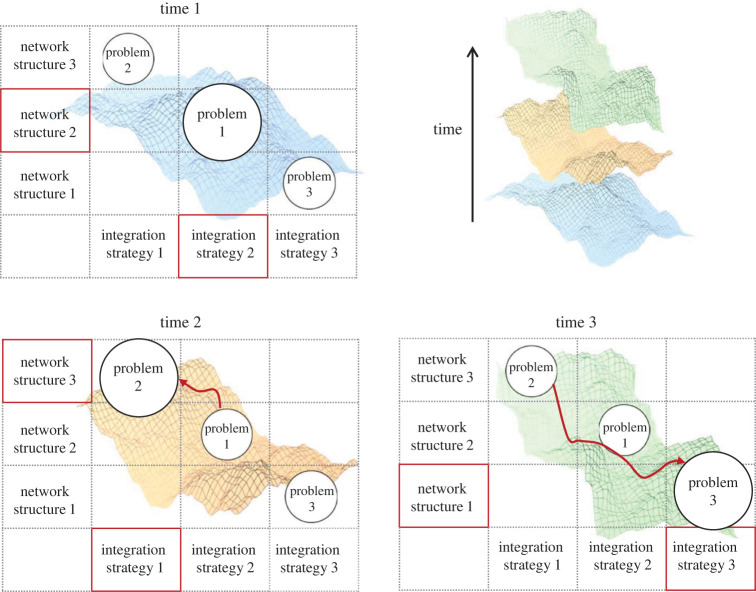
A simplified example of collective adaptation, where a collective ‘floats’ on the adaptive landscape defined by the possible social integration strategies, social networks and problem structures. Over time, the landscape changes: different problems emerge and become more or less important to solve. Collectives adapt towards a set of strategies and networks best suited for the problems faced at a given time. The adaptation can take longer when the new landscape requires a very different set of strategies and networks than the collective was adapted to using before.

Here we argue that, to understand these phenomena and contribute to their solutions, social scientists must better understand the way we collectively adapt to our changing world. We define this *collective adaptation* as dynamic interactions of social integration strategies, social environments and problem structures in complex socio-cognitive systems (figures 1 and 2). Collectives navigate the ever-changing adaptive landscapes resulting from these interactions and adjust their strategies and network structures to the current constellation of problems. As new problems emerge and become important, collective adaptation can take very different and sometimes unanticipated trajectories. While collectives can be well adapted to one set of problems, when the landscape changes they can perform less well than if they were not adapted in the first place. For example, collectives adapted to stable environmental conditions often fail after a sudden change [9], and groups that work well in a small-scale, ‘start-up’ setting often struggle when they need to graduate to a large-scale organization [10].

This adaptation approach goes beyond the current focus on evaluating performance of collectives with a particular constellation of social integration strategies and network structures, typically solving one problem at a time in a static payoff landscape. As epitomized, for example, in phrases like ‘collective intelligence’ and ‘wisdom’ or ‘madness of crowds’, the evaluation approach focuses on rating groups using specific strategies or network structures as more or less intelligent, depending on the number of different tasks they can solve or their accuracy on a particular task [11,12]. However, in many situations of any real-world complexity, optimal solutions can be difficult or impossible to define [13,14], and there are no fixed attractors to which a collective should converge. Collectives instead often grapple with many problems simultaneously, each requiring a different set of strategies and network structures. Adaptive landscapes and the resulting attractors change over time as new problems emerge endogenously and exogenously. As pointed out by Gupta & Woolley [15], more than optimizing performance on any particular task, it is important to understand the processes through which collectives adapt to different tasks and contexts.

Our call echoes, on the collective level, Herbert Simon's earlier individual-level call to go beyond studying outcomes of rational thought in relatively simple, static problem situations [16,17]. We propose to move towards a better understanding of multi-scale, dynamically changing interactions that can drive collective trajectories in sometimes unexpected directions. Our approach builds on the foundation of social and ecological rationality [18,19] and goes further to study the processes of change and adaptation in complex cognitive-social systems.

We build a conceptual framework for studying collective adaptation that synthesizes relevant knowledge across many thematically overlapping guises in cognitive science, sociology, evolutionary anthropology, biology, economics, organizational science, computer science and statistical physics (figure 1, boxes 1 and 2). The framework helps to understand the emergence of collective adaptation as dynamic interactions of its building blocks (figure 2) on different time scales (box 3). We provide a selective review of the findings relevant for the different building blocks and their interactions obtained in different disciplines, and outline several modelling approaches that can be used as starting points for a rigorous, quantitative study of collective adaptation. We discuss implications of the conceptual framework and describe novel research questions of both scientific and societal significance that can be investigated through this lens.

Box 1. **Overlapping views of collective adaptation.**

Aspects of collective adaptation have been studied in different disciplines under different names:

**Collective intelligence.** Also occasionally called ‘swarm intelligence’, collective intelligence has a long history [20] and is extensively studied today. Prominent definitions include ‘groups of individuals acting collectively in ways that seem intelligent’ [20] and ‘the general ability of a group to perform a wide variety of tasks' [12]*.* More recently, researchers have identified different collective processes underlying collective intelligence, such as transactive memory, attention, and reasoning systems [15] and skill congruence [21] which provide groups the capability to better translate individual ability into collective performance [22]. Collective intelligence has been studied extensively in organizational science [23–25], cognitive science [11,26–28], business [29,30], managing intellectual capital [31] and crisis management [32,33], among other fields. This approach also underlies efforts in statistics and computer science to use the power of ensembles [34] and distributed computation [35]. Emergence of collective intelligence has been modelled using analogies from different fields, from statistical physics [36] to neuroscience [37].

**Social learning.** Humans and other animals often learn from others [38–40]. This can be a less costly way than asocial learning to acquire valuable information about good solutions to a variety of tasks, from finding food and mates to solving complex technological and social problems. Many of the strategies described in the social learning literature have also been described in the literatures on collective problem-solving and group decision-making [39–41]. A related body of literature is that on advice taking [42,43], investigating in which circumstances people choose to follow the advice, follow their own mind, or do something in-between.

**Collective problem-solving.** A large literature in behavioural ecology and evolutionary anthropology investigates how humans and other animals accumulate information that no single individual can acquire on their own. For example, relatively simple movement rules allow group members to reach a consensus about the timing and spatial direction of movement [44]; naive individuals can be successfully led to a new food source or migrate in the correct direction [45]; collective movement can average the preferences of the individuals in the group [46] and allow groups to respond to environmental gradients that group members are not able to sense individually [47], in both cases improving the quality of the resulting decision, and animals in groups can discover new routes that individuals cannot [48]. This collectively generated knowledge, when retained at the individual level, is known in biology as ‘collective learning’, and represents an emergent form of social learning [49]. In cognitive science, collective problem-solving has been studied in modelling and experimental studies in the context of decisions from experience [50,51] and exploration–exploitation trade-offs [52,53].

**Group decision-making.** This broad field at the intersection of psychology, management and applied mathematics [54] has contributed a vast array of theories and findings on interacting groups, from research on group decision-making algorithms [55–58] to the value of exchange of information [59]. Group decision-making has also been extensively studied in biology [60].

**Wisdom of crowds.** When and why does the combined judgement of multiple individuals outperform the average and sometimes the best individual in the group? This question is closely related to those asked in collective problem-solving and group decision-making literatures. Studies of ‘wisdom of crowds’ phenomena range from early seminal essays of Condorcet [61] and Galton [62] to recent studies in judgement and decision-making [63], cognitive psychology [64], political science [65], forecasting [66], economics [67], law [68], sociology [69] and biology [70]. Topics of study are the value of experts [71–73], diversity [63,74] and adaptive network structures [75].

**Game theory.** The tools and insights developed within this very broad field are relevant for collective adaptation. In particular, evolutionary game theory is a framework that can be used to model how social integration strategies and networks adapt to task environments over time [76,77] and how the outcomes of evolutionary games depend on the structure of multi-layer networks [78]. Another game theoretical perspective that provides a good starting point to begin understanding collective adaptation are game theoretical models of social dilemmas and problems of collective action [79,80]. For example, Gonzalez *et al*. [50] used the Prisoner's Dilemma to advance the cognitive theory of decisions from experience from individual to dyads, and Moreira *et al*. [81] showed how social networks and collective problems co-evolve.

**Group minds.** Not only do we process information in groups, but the group itself is a rich information processing system that is often much greater than the sum of its parts [82]. For example, work on transactive memory looks at how information is stored within and flows between different members of the group, enhancing the amount of information the group can store and leverage in complex problem-solving tasks [83,84].

**Belief dynamics.** Diverse disciplines have been studying beliefs change over time and on social networks, developing a number of analytic and computational models of belief dynamics [85–90]. These models can help understand why in certain societies new beliefs—such as opinions on climate change or vaccines—spread more quickly than in others, sometimes leading to polarization and other times to consensus, and occasionally leading to backlash effects.

**Evolutionary anthropology.** Several approaches use explicitly evolutionary models to study collective behaviour and changes, typically on a theoretical level and on longer time scales. Researchers have taken a number of different perspectives. For example, *cultural evolution* uses analogies with Darwinian evolution to study cultural change [91] and how changes in human societies accumulate and build on each other [92]. Social learning mechanisms are an essential element of cultural evolution, and studying various forms of social learning has been a theoretical and empirical focus for the field [93]. *Behavioural ecology* focuses on the analysis of fitness benefits of different behaviours and cultural practices. By assuming that humans and other animals aim to maximize their fitness, which can be defined in different ways, studies in this field aim to understand how particular socioecological contexts lead to different kinds of behaviours, including different mating and parenting strategies, cooperative and competitive strategies, etc. [94]. *Cultural niche construction* aims to explain how people adjust the composition of their social networks and their institutions to foster beneficial collective behaviours such as cooperation [95,96].

Many other literatures are relevant for understanding of collective adaptation, but space constraints prevent us from describing them in any detail here. These include but are not limited to crowd psychology [97], collective behaviour [98], social contagion [99], information cascades [100], social diffusion [101,102], herd behaviour [103], social choice [104], forecasting [105], jury decisions [106] and peer production [107].

Box 2. **Parallel efforts.**

Because different aspects of collective adaptation have been studied in relatively isolated disciplines, basic concepts and principles are often rediscovered.

**Frequency-dependent social integration strategies.** Many disciplines have studied frequency-dependent social integration strategies over the last few centuries, from early work in political science [108,109], statistics [110], psychology (conformism; [111]), to economics [112], law [113], organizational science [114], cultural evolution [115], animal learning [116], computer science [117], statistical physics [118], biology [119] and sociology [85]. For example, ‘complex contagion’ models that are rightfully suggested as more appropriate than ‘simple contagion’ in many situations can be viewed as frequency-dependent rules that have been studied in other disciplines under different names [120].

**Majority rule.** While each discipline has contributed useful insights, some basic statistical regularities have been rediscovered a number of times. One such regularity has been described already by Condorcet in 1785: groups using a majority rule can outperform any individual member in finding the correct option, with group accuracy increasing with group size. This can be represented as a cumulative probabilistic process, as described by [121] and [122]; see [123]. However, this work was largely forgotten in social sciences until the second half of the twentieth century [124], when the same relationship between group size and accuracy was rediscovered in cultural evolution [115], psychology [57], sociology [85], biology [119] and statistical physics of belief dynamics [118]. Further, more counterintuitive findings such as that smaller groups can in some circumstances perform better than large groups on tasks with discrete options have also been obtained independently in political science [124], biology [125] and cognitive science [11].

**Belief dynamics.** Similarly, studies of belief formation, change and spread are conducted largely independently in disciplines ranging from social and cognitive psychology, evolutionary anthropology, political science, sociology and computational social science, economics, philosophy and logic, to statistical physics and computer science (e.g. [90,126–132]). Some of these disciplines focus more on social learning processes underlying belief dynamics, others on the structure of social networks on which the dynamics occur; some disciplines study models that are more qualitative and others more quantitative; some employ empirical research while others focus on theory. The same outcomes such as consensus, polarization, fragmentation and zealotry have been studied from different perspectives, with none of the disciplines alone understanding the full picture of the underlying complex socio-cognitive system that gives rise to belief dynamics.

**Network effects.** Parallel efforts in different disciplines can also contribute innovative new insights. For example, social network structures and their implications for individual and collective outcomes have been studied in sociology and social psychology for a long time. With the increased ease of measuring and modelling various phenomena on social networks, researchers from other disciplines such as statistical physics and computer science made a number of additional contributions, revisiting biases due to homophily such as false consensus [133,134], describing novel implications of the friendship paradox [135,136], and extending work on small worlds and degrees of separation [137,138].

Box 3. **Time scales of collective adaptation.**

Collective adaptation can emerge at *evolutionary, historical and contemporaneous time scales*. In *evolutionary* time, collectives of humans and other animals have evolved adaptations that enable quick reactions to life-threatening situations. For example, many animal collectives can detect and react to predators through relatively simple rules of alignment which might have evolved partially as a response to predation threat [139]. Human collectives too exhibit fairly common changes in cognitive processing and network structure when faced with a real or perceived group threat, including rapid collective coordination of beliefs and behaviours, increased altruistic tendencies towards in-group members, and an increased tendency to follow a group leader rather than explore possible solutions on one's own [140,141], which might have been adaptive in situations of real outgroup threats that posed a strong selective pressure in our evolutionary past [142].

In *historical* time, human collectives have been adapting to perpetuating social problems through informal and formal institutions, social norms and cultural artefacts, which provide a default for social integration strategies, network structures and problem representations appropriate in different situations and domains of life [96]. Many societies have developed institutions and organizations dedicated to managing common goods, resolving conflicts and curbing violence, regulating mate choice, electing leaders and regulating private property [143]. Cultural and organizational norms impose further constraints on how individual members can communicate and make decisions. For example, some societies are more likely to follow an established leader's opinion, while others are more prone to establishing consensus [144]. Some collectives maintain open lines of communication between all members of the society while others prohibit communication between certain parts of society (different castes or genders). Voting systems from direct democracy to electoral college to authoritarianism constrain how much individuals can participate in collective decisions. And, societal artefacts from vocabulary and number systems to stories and monuments can strongly influence whether a society finds something problematic and how it represents possible solutions.

Finally, collectives explore possible pathways *contemporaneously*, often starting from evolutionary and historical defaults, but also by reasoning, learning and innovation throughout individual and collective lifespans to make sense of current circumstances and possible opportunities for solutions. Much of the current research in collective adaptation, from organizational science and wisdom of crowds to collective intelligence and animal learning, studies how groups explore task landscapes and decide on best ways to move forward. The *contemporaneous* time scale is the focus of this review.

## 2. Building blocks of collective adaptation

In our framework, collectives are represented as complex adaptive socio-cognitive systems [145] that continuously adjust themselves and their environments in response to different problems. Such complex systems cannot be reduced to a set of individual components—they are wholly entangled, exhibiting what Wimsatt [146] called interactional complexity. However, many aspects of these systems—social integration strategies, social networks, problem structures and adaptation processes—have been studied in separate bodies of literature and developed largely independently in different scientific fields (figure 1, boxes 1 and 2). It is therefore useful to first review what is known about each of them in turn, integrating perspectives from different disciplines, before synthesizing them as a socio-cognitive system that gives rise to collective adaptation.

### 2.1. Social integration strategies

Members of collectives often have different beliefs, including different assumptions about states of the world (e.g. whether vaccination is safe), views on moral and political issues (e.g. whether abortion is a matter of personal choice), evaluations of different issues (e.g. whether a particular politician is trustworthy), preferences (e.g. between different ways to tackle climate change) and goals (e.g. increasing their own wealth and prestige). To take stock of their social worlds, understand current social norms, anticipate others' actions and make decisions, individuals and collectives use different social integration strategies, or cognitive and social algorithms designed to integrate information about beliefs, behaviours and intentions in social environments [39–41,147,148]. These strategies form the interface between individual cognition and social networks, providing information needed for the selection and implementation of other social cognitive strategies, from coordination [149,150], cooperation [151,152] and exploration [52,53], to network building and revision [153]. These strategies can be learned individually, influenced genetically, developmentally and culturally to various degrees, and can be used intentionally or without much conscious thought.

Social integration strategies have been studied in different fields under many different names: social learning strategies (in psychology, cultural evolution and behavioural ecology), belief updating strategies (in economics, sociology, computational social science and statistical physics), group decision-making rules (in psychology and organizational science), voting procedures (in political science and law) and aggregation procedures (in computer science). Despite different names, most of these social integration strategies belong to *several basic classes* that share common principles, information requirements and statistical properties. These commonalities have often not been recognized across disciplines, resulting in sometimes inefficient parallel efforts (box 2). Recognizing these classes is illuminating even though many of these strategies can be modelled as instances of more general processes of Bayesian learning [154,155] or reinforcement learning [156,157].

One large class includes *frequency-dependent strategies* to update beliefs and make decisions based on the number or fraction of others who have a specific belief. Prominent examples are majority, unanimity and minority strategies. These studies have shown that, depending on the threshold for making a decision, frequency-dependent strategies show different signatures of belief dynamics [128] and cultural diversity [158], and have different consequences for accuracy of individual beliefs and consensus formation [57,159,160].

A second class includes *averaging* strategies, whereby information about others' beliefs and behaviours is averaged, either with or without differential weights for specific individuals [62,101,131]. Frequently studied strategies for advice taking, including compromising between own and others' beliefs, keeping one's own or adopting another's belief, can be seen as averaging rules with different weights to self and others [43,155]. Many other rules underlying collective phenomena are essentially also averaging rules in the long run (over time), such as voter rule and Ising models in statistical physics [128], blending inheritance in cultural evolution [115] and contagion rules in epidemiological models of belief dynamics [161].

A third large class of social integration strategies for belief updating includes *model-based strategies* that use different properties of observed model agents, such as their success in a particular task or overall, their expertise, official rank, prestige, closeness, similarity or expressed confidence [40]. Unlike the strategies in the previous two classes, these strategies give zero weights to most of the available models and focus on a few that seem most appropriate for a given problem. While, in principle, these models can also be operationalized as averaging strategies where all but one or a few models have zero weights, the cognitive assumptions behind them are different. In averaging strategies everyone in the sample presumably receives at least some attention, but in model-based strategies most others are not even considered. For example, there is robust evidence that people and other animals tend to copy the beliefs and behaviours of those others who have high social status [162], are well-liked [163] and are similar to the observer [164]. Confidence is another important property of models: confident eyewitness testimony is weighted more by members of a jury [165] and confident people are more influential and trusted [166].

Integration strategies from different classes can be combined, such as when different classes of strategies are used for sampling among social contacts (e.g. by similarity; [75,167]) and for integrating information from the sample (e.g. by a frequency-based or model-based strategy; [168]). In addition, individuals can use diverse state-based strategies [40], including confidence in own judgements [169] and amount of personal information [170] to decide whether to use social information in the first place.

### 2.2. Social environments

Social environments can be conceptualized as consisting of social networks as well as of various social artefacts that human societies have developed to communicate and cooperate, including a variety of languages and scripts, modes of communication from face-to-face to social media, oral traditions, physical records of social knowledge from books and videos to monuments and art, and a variety of intangible and tangible institutions that regulate human relationships, from cultural norms to laws [80,163]. While the description of all of these artefacts is beyond the scope of this review, they provide important opportunities and constraints for how collectives can communicate and adapt. For example, the overall social context can affect whether others might express their opinions truthfully or with some hidden bias, as is often the case in competitive situations and when it is important to assort with similar individuals in diverse populations [171].

Different aspects of social environments can affect collective adaptation by influencing which social integration strategies are more successful, supporting or inhibiting the receipt or acceptance of particular information, and supporting or inhibiting the spread of different adaptive responses. In turn, social environments continuously change in response to demands of the current problem, past experiences with social contacts, and own social cognitive strategies [75]. Collectives can choose to preferentially include or exclude members based on their demographics, expertise, confidence, past cooperative behaviour, perceived membership in an ingroup versus outgroup, or other factors [172,173].

Here we focus on social networks, conceptualized as the set of other individuals generating social information relevant or at least available for the particular problem. Numerous characteristics of social networks have been studied, and excellent reviews exist elsewhere (e.g. [153,174]). We will mention only four considerations that seem particularly relevant for the process of collective adaptation.

One consideration is the difference between perceived and actual social networks. While parts of social networks can be measured ‘objectively’ by using data on who meets and talks with whom, what eventually matters for explaining how social environments affect beliefs and behaviours is the way these environments are subjectively represented in individual minds [175]. Social interactions that are actually relevant to a particular collective for a given problem will depend on what people attend to in the moment, their past experiences and their overall social context [176]. Different people can experience the same social network structures differently, depending on how much they like their social contacts [177,178] and whether they perceive others as members of their group or as outsiders [179]. Similarly, teams with the same objective network structure will have sometimes better and sometimes worse performance when instructed to think of their team as more interdependent [50,180] and to pay more or less attention to others' solutions [25].

Another important consideration is *size and connectivity* of social networks, because these properties affect the size and composition of social samples people can obtain [181]. While larger groups often support greater innovation and cultural complexity [182,183], sometimes smaller groups can make better decisions and be more resilient [70,184], and less well-connected networks can promote collective performance on complex problems and truth finding [185–188]. Interdependence of group members in terms of payoffs and shared information further affects collective performance and cooperation [25,50,180,189,190]. Measures of node centrality such as betweenness predict the spread of information in societies [191].

A third important consideration is *homophily*, the phenomenon that people are typically surrounded by similar others [192] because of self-selection, mutual influence and/or common circumstances [193]. This affects the samples people receive from their social environments and can produce apparent cognitive biases such as false consensus or false uniqueness [194]. Because of homophily in their social environments, and especially if they are using frequency-based strategies for integration of social information, people sometimes think of their current status quo as normal and difficult to change [195], and may try to fit in by adjusting to the perceived norm [196] or be less inclined to support policies that would change the situation [197]. Homophily is also important for the emergence of cooperation and collective action [198,199].

A final consideration we highlight is the fact that one's social contacts are, on average, better connected than oneself (the so-called *friendship paradox*, [135,200,201]). Because of this, one's social contacts are more likely to be both observed and copied, spreading their beliefs and behaviours to others, and affected by new contagious trends themselves. This phenomenon both reinforces the value of using one's social contacts as early social signals of what is about to become popular, and triggers a feedback loop whereby copying these valid signals reinforces social contacts' influence on future trends.

Despite the obvious importance of social networks, disciplines differ in the extent to which their models of collective behaviour explicitly include network structure. While the study of social networks has a long and fruitful tradition [153,202,203], in much of psychology and behavioural economics, partially due to the constraints of laboratory-based experiments, studies have typically focused on small, fully connected groups, or have used descriptions (e.g. vignettes) rather than direct interactions with social environments [204]. In cultural evolution, networks are often conceptualized as different routes for transmitting information, though they have only recently been included in formal models [183,205]. Instead, researchers more broadly use age as the feature structuring the population and refer to parent-to-offspring transmission as ‘vertical’ transmission, peer-to-peer transmission as ‘horizontal’ transmission and intergenerational transmission that does not involve parents and offspring as ‘oblique’ transmission [206]. Who people learn from shifts across their lifespans: children are likely to learn from parents and caretakers, while adolescents and adults are more likely to learn from peers and experts [207–209]. These disciplinary differences have been recently blurring with the fast-developing field of computational social science, where interdisciplinary teams study a variety of processes on complex and adapting network structures in web-based studies with human participants and in computational models [210].

### 2.3. Problem structure

Collectives and the individuals within them face multiple problems at any given time, from avoiding various dangers, developing technological solutions, to organizing and coordinating social relationships. These problems can occur both exogenously from the outside environment, and endogenously from the coevolution of integration strategies and social environments in response to past problems. For example, modern communication technologies have enabled the development of novel integration strategies (such as various rating systems) and social networks (larger and easier to change) that speed up the exchange of useful information. At the same time, these solutions have been a source of many emerging novel problems, such as the spread of misinformation and disinformation, the development of echo chambers and polarization. Attempts to deal with these problems involve inventing novel strategies and network structures that in turn are likely to lead to further problems.

A complete characterization of possible problem structures would require a complete representation of the world around us, which is impossible. However, it is useful to distinguish between problem structures that require different social integration strategies and social networks, in particular because many existing papers focus only on a single problem. We provide three examples of such important distinctions that show the importance of comparing several problem types within the same study. One distinction is between problems involving categorical versus continuous judgements. In political science, collective decisions are often studied in tasks that involve a choice between two or more discrete options, such as voting for different candidates [108]. By contrast, much of the ‘wisdom of crowds’ literature in psychology, economics and other disciplines typically uses tasks involving continuous judgements, such as guessing the weight of an ox or future inflation rate [211]. It is often assumed that the wisdom-of-crowds findings apply also to discrete choices, but while this is true for some of the findings (e.g. that group diversity is typically beneficial for group performance for both kind of tasks, [159]) it does not hold for others (e.g. that larger groups always outperform smaller groups; [124]).

Another important distinction is between simple and complex task landscapes. In organizational science and anthropology, and recently in economics and cognitive science, researchers often study problems whose solution space resembles rugged landscapes: local maxima can sway one away from an even better solution elsewhere in the problem space [212,213]. This matters because tasks that involve simple payoff landscapes with one dominant solution benefit from highly interconnected social networks and fast social learning rules. By contrast, tasks that involve rugged landscapes with many local optima can benefit from slower networks and from more individual exploration versus social learning [167,168,185,187,214,215].

A third distinction is between one-shot and repeated problems. In most disciplines, collective performance is studied in one-shot problems. However, most problems unravel over time, with payoffs of different options changing more or less predictably over different time scales. The field of dynamic decision-making, a subdiscipline of cognitive science, has focused on these types of tasks, showing for example that individuals and groups with shorter memories and noisier copying strategies can be more successful over time in changing environments [50,51]. Other important distinctions include the way rewards are split among the members of the collective [80], distribution of relevant information [216], predictability [217] and speed of environmental change [218].

Beyond the properties of the specific tasks, problem structures are also defined by more general properties of the global environment that constrains the type and usefulness of possible strategies, networks and tasks. Economic and political factors as well as culture and prevailing societal norms are all important sources of these global environmental constraints. For example, some cultures promote the use of frequency-dependent integration strategies such as the majority rule, while others might prefer following a particular individual. In other cultures, social networks cannot exclude certain genders or ethnicities for some problems. And in yet others, problem structures do not include solutions that might be considered elsewhere, such as restricting free speech or the right to bear arms.

## 3. Emergence of collective adaptation

Now we are ready to synthesize social integration strategies, social networks and problem structures into a complex adaptive system in which these components dynamically interact and adjust to each other. We can visualize this process of collective adaptation in a simplified space of integration strategies, networks and problems, all evolving together in response to each other over time (figure 2).

In this cartoon example, a collective is facing three problems, each best solved by a different combination of integration strategies and network structures (as indicated by the position of problems on the landscape). The underlying payoffs for solving different problems (the adaptive landscape) change at each time point. At time 1, problem 1 is the most important for most individuals in the collective and solving it brings the collective to the peak of the adaptive landscape. The collective will therefore tend to adjust their integration strategies and network structures in ways that enable good performance on problem 1, even if this adjustment impairs performance on the other two problems. For example, a collective might be focused on developing complex technologies and may benefit from relatively loose network structure with occasional exchange of information to find the currently best solution [187]. At time 2, the underlying adaptive landscape has changed either because of external environmental changes or endogenous dynamics, and problem 2 now becomes the most important. The collective adapts individual integration strategies and network structures towards those combinations that enhance performance on problem 2. For example, another group might attack the collective, which now has to adapt towards rapid mobilization and unwavering obeyance of a few leaders. This problem usually benefits from a cohesive society with a hierarchical structure that enables rapid transmission of information and commands [219]. At time 3, the landscape changes again, with problem 3 becoming the most important—for example, adjusting to sudden changes in climate. Now, the collective needs to adapt towards a quite distant set of network structures and integration strategies, enabling both strong local cooperation and global coordination. Over time, the collective ‘floats’ in this space depending on the current problem structures faced by its members, as well as on their available integration strategies and network structures. Similar scenarios can be imagined for collectives of different sizes and purposes, from families to teams and organizations, to countries, and adaptive changes can occur on different time scales (box 3).

Of course, in reality, this adaptive landscape is high-dimensional and ever-changing. While in the caricature example in figure 2 we presented integration strategies and network structures as each varying along a single dimension, there are in fact numerous dimensions on which strategies and networks differ. In this high-dimensional space, it might not be necessary to cross ‘valleys’ of low fitness in order to reach a more adaptive state in the landscape. Rather, collectives could use shortcuts (fitness ridges) to move from one fitness peak to another [220,221]. In addition, the adaptive landscapes are constantly changing due to their endogenous dynamics and external factors, and thus are more reminiscent of ‘seascapes’ [222]. Nevertheless, the landscape analogy can be a useful starting point for thinking about collective adaptation, provided that one is aware of its limitations [223,224]. Even if collectives do not need to get stuck in any part of the landscape (or seascape), this analogy makes it clear that knowing the past trajectory of a collective is crucial for understanding its adaptation (or lack of thereof) to new circumstances. It also helps to understand other relevant aspects of collective adaptation, including the impracticality of optimizing for a single problem, collective myopia and the value of pre-emptive exploration, as we discuss next.

## 4. Implications of the collective adaptation perspective

While vastly simplified, the example in figure 2 helps to understand *five implications* of the collective adaptation perspective that distinguish it from more traditional ‘collective intelligence’ and ‘wisdom-of-crowds’ approaches focused on collective performance in specific tasks.

### 4.1. Path dependence

Collectives that currently face the same set of problems can take different trajectories in the space of integration strategies and social environments, depending on the past landscapes they have been adapted to. For example, a country with a strong central government will take a different route toward adapting to a pandemic than a country with weaker and localized governmental structures. In contrast with the more traditional collective intelligence approaches that focus on finding a single point in this space that is best suited for a particular problem, the collective adaptation perspective allows for understanding why some collectives can find it harder to adapt to emerging problems. Their societal structures, belief systems and problem-solving strategies might have been adapted to very different challenges in the past, and adaptation towards new constellations can be difficult because of individual and institutional inertia, lack of relevant knowledge and skills, and high costs of reorganization.

### 4.2. No single ‘intelligence’ dimension

A collective adaptation perspective does not assume a single latent dimension of ‘collective intelligence’ that would explain a collective's superior performance on many tasks. Rather, it allows for explaining correlations in collective performance as occurring from dynamic interactions of initially unrelated integration strategies and social environments co-occurring over time (akin to explaining correlations in individual performance on intelligence tests by the dynamic interaction of intra-individual processes over time; [225]). Consequently, the same measured level of collective intelligence—the general latent factor discovered in correlational analyses of behavioural outcomes (as in e.g. [12])—might occur from an interaction of different combinations of underlying cognitive and social processes. The particular combination will depend on the historical trajectory of a particular collective. Because of constant adaptation, it is also possible that a group that was ‘intelligent’ at one time point later becomes less ‘smart’ (see section 6 on emerging research questions for examples).

### 4.3. No optimization for a single problem

Current collective behaviour might not be ‘optimized’ to perform well on any one of the problems it is facing. Instead, collective performance reflects individual and group attempts to perform well enough on at least some of the encountered problems, with different individuals possibly focusing on different problems, and with the repertoire of problems and available strategies and network structures constantly changing. Therefore, collectives can seem ‘stuck’ in suboptimal behaviours when judged by a single criterion that seems most important to a ‘rational’ outside observer. For example, collectives can persist with environmentally damaging practices or ethnic discrimination even when these practices seem overall detrimental. In fact, these collectives and individuals within them might be balancing several problems at the same time, such as maintaining system stability and individual security through adhering to existing social norms and hierarchies.

### 4.4. Collective myopia

The constellation of social integration strategies and networks that are best suited for a particular problem might not be immediately obvious or available to the members of a collective used to solving a very different set of problems. For example, even though a particular strategy for integrating social information—for instance a democratic, frequency-based strategy such as following majority—might be best suited for collectively solving today's complex problems, many societies without direct experience in democracy struggle to establish and maintain it. Collectives sometimes need to cross suboptimal stepping stones on the way to discovering better constellations and can be prone to returning to older, known constellations when this adaptation phase becomes difficult. For example, young democracies with recent experiences in autocratic systems may be prone to returning to those systems [226].

### 4.5. Benefits of exploring alternative social worlds

It can be beneficial for collectives to explore the space of possible strategies, networks and problem structures, so that when the environment changes the collective can adapt in the right direction faster. This might be one of the reasons why humans, individually and collectively, often engage in behaviours that appear to not have a particular goal, such as various social games and numerous forms of storytelling (from myths and epics to theatre and cinema) aimed just as much at describing the current world as at exploring hypothetical alternative worlds. Among other benefits, these activities enable exploration of the space of possible solutions and rehearsing collective responses to different situations, enabling faster collective adaptation in times of quick, significant changes.

## 5. Studying collective adaptation

### 5.1. Existing findings on interactions of building blocks

Many interactions between the building blocks of collective adaptation have been documented in different, largely disconnected literatures. To foster transdisciplinary integration of this knowledge in our overall framework, we review findings of their dyadic and triadic interaction patterns. Further studies of collective adaptation can build on these existing findings. Each of the findings in this section can be seen as one point on the complex landscape that collectives must navigate. A main question for further research is to understand and model how collectives traverse between these different points.

#### 5.1.1. Interaction of social integration strategies and problem structures

People can flexibly adapt their integration strategies to the requirements of a particular task [40]. Humans and other animals show a continuum of flexibility, from facultative switching in response to developmental cues (e.g. learning socially when pregnant to avoid the risks of asocial learning, or learning from peers rather than parents when early developmental environments contain markers of stress) and social network position [227], to the individual learning of social learning (e.g. learning via reinforcement that copying others for particular problems is beneficial) and the social learning of social learning (learning from others how to learn from others; [228]), which may lead to cultural variation in the use of social learning strategies [229,230]. Reward experiences in a particular task can promote more or less social learning, with individual exploration being more pronounced at early stages of collective problem-solving and with declining payoffs, whereas exploitation of previous solutions is more likely later on and when payoffs are good [37,231–233]. In turn, this affects individual and group performance in fast-changing environments, where individual exploration is more useful than copying past solutions [51]. Cognition can also adapt to specific tasks that have been particularly consequential in our evolutionary past, making some concepts easier to learn and transmit in a group [234–237]. For example, urban legends or gossip that contains survival, social or emotional information are transmitted better than those that do not [238,239].

While social integration strategies can be adapted to the task at hand, the best strategies for a given task may not be available to all collectives or all individuals within a collective because of historical and cultural constraints. For example, organizations and governments might be designed to integrate information in a particular way (e.g. through a set of more or less democratic institutional structures) which can be difficult to change. Such entrenched integration rules can lead to problems of their own making. For example, an organization where decisions are made by a central authority can find it difficult to deal with problems requiring diverse opinions. A society facing a problem that requires collective action but whose decision structures emphasize individual and minority rights may find it difficult to build the consensus needed to solve the problem.

#### 5.1.2. Interaction of social integration strategies and social environments

The interplay of social network structures and different integration strategies can significantly change the dynamics of belief spread and collective adaptation [240]. For example, the fitness of different cooperative strategies [241] and social learning strategies [242] is highly dependent on the distribution of competing strategies in the population. Inference strategies that produce high variance in individual solutions perform better when used in groups than when individuals use them alone [217]. Diversity of solutions in general promotes collective performance [74,243]. Integration strategies and social environments interact so that strategies relying on less social learning perform better in more connected networks, and those that rely on more social learning perform better in less connected networks [167,214,244].

Even more than social integration strategies, social networks can be difficult to change because of physical infrastructure, cultural and historical reasons, and familial and financial connections. Collectives can be stuck with particular network structures that can make solving novel problems difficult. At the same time, collectives might experience exogenous pressures on network structures, from abolishing or establishing segregation laws to the growth of social media technologies. These changes can require novel social integration strategies, such as learning new metacognitive cues for recognizing true and false information coming from novel social environments [245], and appropriately judging cues such as confidence [244,246] or past performance [155] to decide how much to take into account others' opinions [247].

#### 5.1.3. Interaction of social environments and problem structures

For complex tasks sparse social networks in which information spreads slowly typically lead to better collective decisions [185,187], whereas for simpler tasks well-connected networks perform better. Smaller groups can be more accurate than larger groups when making a series of categorical decisions [11,70,125], but large groups are typically better than smaller groups when it comes to making accurate continuous judgements [74]. Interacting groups can achieve better outcomes on tasks that require pooling of unshared knowledge [59]. While problems can shape networks, changes in social networks in turn can produce a host of new unanticipated problems [3], from misinformation and polarization to conflicts and inability to solve collective problems.

#### 5.1.4. Interaction of social integration strategies, social environments and problem structures

In addition to the dyadic interactions described so far, all three components interact and adjust to each other. For example, in response to problems such as outgroup threat or increased uncertainty, collectives might change integration strategies from democratic to more authoritarian and at the same time tighten up their networks to purge perceived intruders and speed up information flow [248,249]. In turn, problems that collectives face can emerge and change depending on integration strategies and network structures incentivized in a given society [96]. For example, increased polarization of opinions can give rise to fragmentation of social networks and reduced social trust, which in turn creates problems related to managing common resources such as climate [250] and solving collective problems such as pandemics [251]. Social environments and problem structures also interact with and shape the effectiveness of integration strategies [252]. To decide which strategy to use in different social and problem contexts, individuals and groups use metacognitive cues about their own and others’ past and likely future performance [247,253]. One such cue can be the speed of response of different group members: those who have better individual information are likely to react first, allowing the others to learn socially from them, which can be particularly advantageous when costs of errors are high or when the group situation is competitive [170]. Strategy choice can also be affected by cultural norms (e.g. following an authority figure might be more valued in some societies, while in others consensus decisions based on frequency-dependent or averaging strategies may be more valued), network properties (strategies such as majority rule require more interconnected networks than some model-based strategies such as following a leader; [254]), and task characteristics (e.g. whether it is more important to find a quick collectively acceptable solution or find the best possible solution; [214]).

### 5.2. Promising modelling approaches

How can we go beyond verbal explanations to model in a rigorous way how collectives navigate the complex adaptive landscapes of integration strategies, social environments and problem structures? Ideally, models of collective adaptation should be as simple as possible to enable understanding and stimulate further theory development even when they are wrong (as ultimately all models are). For example, the landscape analogy and related models obviously miss some important aspects of collective adaptation, but these errors are easy to grasp and can shed light on aspects that need more theorizing. More opaque and complicated models can be more accurate but less conducive to further insights. There is no one best model or analogy for complex social phenomena such as collective adaptation—each analogy will have limits and associated conceptual baggage [224,255]. It is therefore fruitful to explore many different analogies and modelling techniques and compare their predictions of real-world phenomena.

Furthermore, models of collective adaptation should be implemented mathematically or computationally and produce quantitative predictions of future individual and collective trends. Beyond mathematical models, system dynamics models can be used to better understand relationships and feedback loops between different building blocks of collective adaptation [256–258]. Agent-based models allow implementation of diverse integration strategies on the level of individuals embedded in different social environments and problem structures [86,152,259–262]. Computational models should be inspired and—importantly—constrained by theory and evidence about human cognition and sociality.

Models should ideally be able to describe and predict collective performance across different contexts, by finding dimensions along which different tasks, strategies and networks structures can be compared (e.g. task complexity, strategy class, network size and interconnectedness). Models can also go beyond mere performance and try to predict collective ability to adapt. In most real-world situations, collectives need to juggle several different tasks simultaneously in continuously changing environments. Adaptation therefore might be better expressed as the ability to anticipate, cope with and/or reorganize in response to changes in problem structures, strategies and social environments [263], or more broadly as a version of social resilience [264,265].

Finally, as all models will be at least partially wrong, it is imperative to continuously test their assumptions and predictions in real-world situations. One must compare different models that can predict similar trends and explore when and why they make reasonable predictions and when they fail. Fortunately, scientists have never had access to so much empirical data about different aspects of human cognition and sociality as today. It is now more convenient than ever to obtain data from group experiments and observational studies [266], historical and archaeological data, ethnographic data, mobile phone data [267] and other sensing methods [268], to longitudinal surveys [269,270], and analyses of large textual corpora and other by-products of human interactions (e.g. [271,272]). These sources of data, in particular those which include longitudinal information about problems, strategies and network structures faced by a particular collective, can all be used to study collective adaptation.

While describing different modelling paradigms is beyond the scope of this paper, here we mention several paradigms that are good candidates for developing models of collective adaptation.

#### 5.2.1. Evolutionary models

The collective adaptation perspective is fundamentally dynamic and evolutionary in nature. This does not mean that it is focused on the genetic or biological roots of collective behaviour, only that it is focused on how collective systems respond to endogenous and exogenous pressures and shocks. Adaptation need not involve biological reproduction and can be based on social transmission occurring on time scales from moments to many generations [247,273,274]. Evolution can be used as an analogy to help model how collectives adopt different social integration strategies given their collective history, current cognitive and social capabilities, and momentarily attended problem structures [91,115,116,206,275]. Evolutionary models can help reveal how natural selection can lead to effective group-level collective responses, while operating on selfish individuals [276,277]. One aspect of collective adaptation that these models typically lack is network structure, in particular complex structures that occur in the real world. Recent modelling efforts have begun to study the coevolution of network structures and integration strategies [205,278], generalist or specialist problem environments [279] and cooperation [280].

#### 5.2.2. Bayesian reasoning

While fully Bayesian models of social cognition are too computationally intensive to be cognitively plausible, this analogy can be useful to understand existing and develop new social integration heuristics that resemble optimal balance between exploration and exploitation [154,155]. For example, mathematical models of optimal decision-making using Thompson sampling have been successful in modelling cooperative multi-armed bandit problems with varying communication constraints [281,282]. To be useful for studying collective adaptation, these models could be extended to more complex and simultaneous conflicting problems, as well as to other network structures.

#### 5.2.3. Statistical physics

One can also model collective adaptation using analogies with systems of particles as studied in statistical physics. Models based on the statistical physics framework typically involve a collection of agents connected in a particular way, who can choose between different states according to specified rules. Statistical physics models can incorporate psychological concepts[87,283–285] such as cognitive dissonance (energy), uncertainty or lack of attention (temperature), subjective representations of networks (linkages) and belief integration strategies (updating rules). Other physics analogies that have been used to study collective adaptation are percolation [286], diffusion [287], Monte Carlo methods [288] and quantum physics [289]. A related analogy that has been successfully used in many models of aspects of collective adaptation, in particular belief dynamics, is epidemiology [290,291]. To be suitable for studying collective adaptation, these models should involve mechanisms (meta-level strategies) for switching between integration rules and network structures as problem environments change.

#### 5.2.4. New paradigms for modelling collective adaptation

Because collective adaptation involves an interaction of cognition, social environments and problem structure, modelling it is likely to require connecting several frameworks, each useful for a particular component or time scale. One example is Tump *et al*.'s [170] combination of drift-diffusion cognitive models of individual and social learning with an evolutionary process that selects parameters of drift-diffusion models that are best adapted to particular group structures and problems with different costs of errors. Another example is Cooney *et al*.'s [292] work on modelling the evolution of sociality that combines epidemiological models, a replicator equation and adaptive dynamics models to study evolution on three different time scales. We believe that successful modelling frameworks will involve such combinations of models suitable for different aspects of collective adaptation. Computational models can be particularly suitable for such integration.

## 6. Research questions emerging from the collective adaptation framework

The collective adaptation framework enables researchers to begin answering a number of critical questions for social science and our society. These questions are difficult to investigate within the collective intelligence framework where intelligence is seen as a fixed attribute of teams in a static problem environment, and where research is often confined to disciplinary silos. The lens of collective adaptation helps to think about these complex problems in a new systematic way. Each of these questions can be reframed as a problem of finding a new combination of integration strategies and social environments after being adapted to a different problem structure in the past. Researchers can use models and empirical studies to simultaneously explore these building blocks of collective adaptation.

### 6.1. Why is it sometimes hard for collectives to reach seemingly obvious solutions to a particular problem?

Researchers and other citizens often wonder why their collectives cannot seem to find solutions to problems such as climate change, mass shootings, racism or pandemics. To many individuals, it seems obvious what needs to be done, but collective trajectories seem difficult to steer in the right direction. The collective adaptation framework offers several insights about possible reasons. One is that collectives try to solve many different problems at the same time, and integration strategies and networks suitable for solving one problem can interfere with solving other problems. For example, collectives might try to maintain stability by preserving existing strategies and network structures, even though those might have been developed for very different problems in the past (e.g. Electoral College in the United States or veto rights in the United Nations). A related reason is that different groups within collectives often have different preferences, and strategies used to integrate these preferences are not adapted to the problem at hand but are chosen because of historical, cultural or power-related reasons. For example, collective decisions such as those about climate change or abortion are made not by a frequency-based strategy such as majority rule, but rather by following specific influential members, chosen because of financial or historical institutional advantages. Finally, collectives can be stuck in an unfavourable part of the problem landscape, where moving in any direction brings temporarily worse performance before any improvement is achieved. For example, dealing with climate change might reduce employment or profits of key members of the collective, making them reluctant to initiate changes that would ultimately lead to better outcomes for all.

### 6.2. Why do some collectives develop successful ways of coping with a specific problem and others seem to fail?

As it became obvious during the COVID-19 pandemic, some countries managed to achieve a high level of vaccination and relatively low rate of deaths, while others did not [293]. Many other problems, from achieving democracy to reducing corruption, can be more or less difficult to solve for different collectives. While the collective adaptation framework does not provide immediate answers, it suggests that a way to understand these problems is by understanding the historical trajectories that the collectives have navigated while solving past problems. In particular, the social integration strategies and network structures that they have developed in the past might be impairing their ability to cope with novel problems. For example, collectives that traditionally engaged in more individual learning and had decentralized networks might be less likely to accept government-enforced vaccination [294].

### 6.3. Why do some successful collectives experience reduced performance over time?

Sometimes, initially high-performing teams become worse over time. Aside from regression to the mean and related issues such as the noisy relationship between performance and true ability [177,178], the collective adaptation framework offers further avenues of inquiry. For example, early success can make a team more tightly interconnected over time [295]—in turn impairing its performance on further complex tasks where diversity of opinions is needed for success [25,187]. And increased cooperation in social networks can support the formation of more long-range social ties, which can create the conditions for the collapse of cooperation [280].

### 6.4. How do collectives change their integration strategies and network structures to adapt to different problems?

The collective adaptation framework points to the crucial need for studying the meta-level strategies that collectives use for these decisions. While, as described before, there is much research on the social integration strategies and network structures that individuals and collectives use to solve different problems, there is little research on meta-level strategies that collectives use to switch between different strategies and networks depending on problems they face. In individual decision-making, it has been shown that individuals can adapt their social integration strategies to the task at hand [296–298], with strategies depending on cognitive abilities [299], time pressure [300], past performance [231] and knowledge of cues [301]. In social contexts, meta-level strategies have been studied in advice taking [155,302] and in teamwork [232], but more research is still needed on how collectives decide to use different strategies [303], and in particular how they decide to restructure their networks (but see [75,279,280]). Answering these questions is further complicated by the presence of social norms and more formal institutions that limit the set of strategies and network structures that collectives can choose from. A promising perspective is the transactive systems framework [15], which describes how collectives use their memory, attention and reasoning systems to sense changes in their environment and coordinate actions. Another promising framework is Bayesian collective learning, based on the mathematical theory of optimal decision-making, which can be used to anticipate and justify different heuristic strategies for social integration and network updating in circumstances of limited and erroneous knowledge about others' strategies and network connections [282]. Finally, group and collective emotions such as perceived threat from an outgroup or group pride can be powerful triggers for quick restructuring of a collective's network structure and the way it integrates social information [304,305].

### 6.5. Can we anticipate new problems that might emerge because of the way societies adapted to past problems?

Collective adaptation perspective suggests that one can anticipate problems that collectives could encounter in the future by understanding what problems they had to solve in the past. Adaptation to previous environments will determine their currently used integration strategies and network structures, and affect the ease with which collectives can adapt to new contexts. For example, more tightly knit collectives and those that follow a few dominant members rather than using frequency-based integration strategies might be more likely to encounter difficulties in solving complex problems that require diverse opinions about possible solutions. As another example, groups experiencing threat can adopt a more homogeneous mindset that is prejudiced towards intruders and allows for quick collective action, but that at the same time, facilitates the spread of harmful beliefs that can endanger the groups in the long run [306]. Of course, predictions of collective adaptation trajectories can be inherently limited by the complexity of the underlying socio-cognitive systems, but it is useful to explore the extent of gains that can be made by theoretical and methodological development.

### 6.6. How can we reduce less desirable consequences of emerging collective problems?

Better understanding of complex adaptive socio-cognitive systems that give rise to undesirable consequences (e.g. spread of misinformation, hate speech, extremism and violence) could ultimately allow collectives to adjust their trajectories in order to reduce the likelihood of such consequences. This would be an improvement to the current societal response to these phenomena, which is focused mostly on alleviating the consequences once they occur rather than preventing them. Ideally, the collective adaptation framework could be leveraged for societal impact in areas such as enhancing or developing cultures of sustainability, promoting beneficial health behaviours and promoting a healthy societal dialogue. At the same time, it might help societies to avoid adversarial attempts to manipulate collective decisions and behaviours.

## 7. Outlook

The collective adaptation perspective goes beyond the traditional idea of collective intelligence, by explicitly acknowledging and modelling several critical aspects of real-world collective behaviour: path dependence, impossibility of optimization, collective myopia and seemingly aimless exploration of alternative worlds. More generally, the collective adaptation perspective does not search for a generally intelligent collective but asks instead, ‘What pathways might collectives take in pursuit of their various goals, given the problems they have been adapted to in the past?’. Understanding the dynamic interactions of integration strategies, social environments and problem structures opens a path towards studying important scientific and societal questions such as why collectives fail to reach seemingly obvious solutions, how they adapt their integration strategies and social network structures to novel problems, and how they can avoid less desirable future pathways. We hope this article will inspire researchers to develop and empirically test rigorous models of collective adaptation based on the rich knowledge accumulated across fields, thus contributing to a transdisciplinary, quantitative and societally useful social science. Only such social science can help people to understand our rapidly changing and ever more complex societies, avoid collective disasters, and reach the full potential of our ability to organize in adaptive collectives.

## Data Availability

This article has no additional data.
